# Transition to Independent Surgical Practice and Burnout Among Early Career General Surgeons

**DOI:** 10.1177/15533506211039682

**Published:** 2021-08-31

**Authors:** Mohammed Firdouse, Caitlin Chrystoja, Sandra de Montbrun, Jaime Escallon, Tulin Cil

**Affiliations:** 1 12366 University of Toronto, Toronto, ON, Canada; 2 60329 Department of Surgery, Toronto, ON, Canada; 3 10051 Princess Margaret Cancer Centre, Toronto, ON, Canada

**Keywords:** general surgery, residency, transition, burnout, stress, independent practice

## Abstract

*Background*: The transition from surgical residency to independent practice is a challenging period that has not been well studied. *Methods*: An email invitation to complete a 55-item survey and the Maslach Burnout Inventory–Human Services Survey (MBI-HSS) was sent to early career general surgeons across Canada. The chi-square test or Fisher’s exact test was used to compare demographic and survey characteristics with burnout. Multivariable logistic regression was performed. *Results*: Of the 586 surgeons contacted, 88 responded (15%); 51/88 surgeons (58.0%) were classified as burnt out according to the MBI-HSS. Most surgeons (68.2%) were not confident in their abilities to handle the business aspect of practice. The majority (60.2%) believed that a transition to independent practice program would be beneficial to recent surgical graduates. *Conclusions*: Our data showed high prevalence of burnout among recently graduated general surgeons across Canada. Further, respondents were not confident in their managerial and administrative skills required to run a successful independent practice.

## Introduction

Within North America, medical trainees must successfully complete a residency program and in some cases, pursue fellowship training before they can start practicing as an attending staff in independent practice.^1^ This transition from trainee to staff physician may be stressful and overwhelming.^2,3^ Within a short period of time, staff physicians are faced with multiple challenges such as adapting to a new setting with new colleagues, taking on the role of primary clinical decision maker, and managing non-clinical tasks such as teaching, research, financial planning, and their personal lives. The issues affecting recent graduates during this stressful transition period have been sparsely covered in the literature, and none of the published studies resulted in an empirically founded conceptual framework.^4-10^ The American College of Surgeons has recognized this and focused efforts toward creating transition to practice programs to better equip residents for a smoother transition.^11^

Recent studies also highlight the growing problem of burnout in medical practitioners.^12^ Within general surgery, rates of burnout are significant and may manifest with symptoms of depersonalization, emotional exhaustion, and reduced personal accomplishment.^13^ Physician burnout is a serious area of concern as it has been correlated with low morale, personal dysfunction, insomnia, physical exhaustion, personal problems, poor performance, and deterioration in quality of medical care.^14,15^ In addition, both depression and burnout have been associated with major medical errors.^16^ Of note, studies have demonstrated that burnout develops as early as medical school and continues throughout residency.^15,17^

Considering the potential increase in stress during the transition from residency or fellowship training to independent practice, we hypothesize that this subset of surgeons may have particularly greater prevalence of burnout.^13^ Furthermore, most residency training programs focus on competencies and skills development in the residency curriculum. This leaves little or no teaching on the other aspects of career management including the administrative and business responsibilities of medical practice. The aims of this research were twofold. First, we sought to assess issues affecting new graduates of general surgery programs within Canada, particularly their perception of their ability to manage an independent practice. Second, we assessed the prevalence of burnout among this subset of general surgeons.

## Methods

After obtaining institutional research ethics board review, a study invite was sent out via email to early career general surgeons across Canada asking them to complete a cross-sectional survey accessible through a provided URL from www.fluidsurvey.com. The names and contact information of surgeons were obtained from the database of surgical graduates participating in the annual General Surgery Review Course in Toronto, ON. This included surgeons who had graduated between 2007 and 2012 from a general surgery residency program in Canada. The survey was composed of 55 questions covering demographic factors, issues regarding independent practice, surgeons’ perception of their roles, responsibilities, and training preparation. We also included the validated Maslach Burnout Inventory–Human Services Survey (MBI-HSS).^18^ Three iterations of the invitation emails were sent with 2-week intervals in between each. The completion and submission of the survey indicated implied consent for the data to be used in the study.

The demographic characteristics section of the survey was constructed by referring to several papers assessing burnout in healthcare providers,^19-22^ especially Soler et al, Balch et al, Napolitano et al, and Friedell et al. The MBI-HSS is the most widely used measure of burnout for healthcare professionals. It has been extensively tested for its reliability and validity since its creation in the early 1980s. Consequently, the MBI-HSS has become the gold-standard tool for burnout assessment and was selected for this study based on its strong psychometric properties. The MBI-HSS is a 22-item self-report questionnaire that measures three dimensions of the burnout syndrome: emotional exhaustion (EE), depersonalization (DP), and reduced personal accomplishment (PA). The response for each question is a 7-item Likert scale ranging from 0 (never) to 6 (everyday). For each dimension, the total score is calculated by summation of its questions and then classified into “low,” “moderate,” and “high” categories based on the cutoff scores outlined in the MBI-HSS interpretation guidelines. There is considerable heterogeneity in how studies define burnout syndrome.^23^ For this study, we classified participants with high EE (score ≥27) or high DP (score ≥13) as having burnout.

We described demographic, survey, and burnout characteristics for all participants. Categorical variables were described as numbers and percentages. The chi-square test, or Fisher’s exact test as appropriate, was used to compare demographic and survey characteristics with burnout. Missing responses for question components related to EE or DP were assigned a score of 3. A sensitivity analysis was conducted assessing the impact of missing response score assignment on classification of burnout.

Multivariable logistic regression of the association of demographic and survey characteristics with burnout was performed utilizing the screening variable selection method. First, we selected covariates with a *P*-value of .25 or less on bivariate analysis, with burnout as the outcome variable. Second, we selected variables with the highest chi-square value, for the greatest number of variables allowed by the convention of one independent variable for every 10 events (or non-events, whichever is smaller). Third, we constructed a full model and verified model assumptions. *P*-values less than .05 were considered statistically significant. All statistical analyses were performed with SAS Studio 3.6 (SAS Institute, Carty, NC).

## Results

### Demographics

A total of 630 early career general surgeons were contacted; 44 (7%) emails bounced back due to inactive or incorrect contact information. Of the remaining 586, 88 participants responded to the survey (15.0%) with a 98% completion rate; 44 (50%) responses were from men, 43 (48.9%) from women, and 1 participant (1.1%) chose not to disclose their sex. The majority of participants were married (68.2%). Most (41.4%) general surgeons had 2–3 years (37.5%) of independent surgical practice (see Table 1 for more details).

**Table 1.** Demographics of Survey Participants (n=88).

Demographics	n	Percentage of total respondents
Age		
25–35	23	26.1%
36–45	58	65.9%
46–55	5	5.7%
56–65	1	1.1%
>65	1	1.1%
Gender		
Male	44	50%
Female	43	49.9%
Undisclosed	1	1.1%
Marital status		
Single	14	15.9%
Partnership	7	8.0%
Common law	5	5.7%
Married	60	68.2%
Divorced/separated	2	2.3%
Years since MD attained		
1–5	7	8.0%
6–10	42	47.7%
11–15	32	36.4%
16–20	4	4.6%
>21	3	3.4%
Specialty		
General surgery	36	40.9%
Breast surgery	3	3.4%
Surgical oncology	13	14.8%
Pediatric surgery	4	4.5%
Trauma surgery	6	6.8%
Minimally invasive	13	14.8%
Other	12	13.6%
Missing	1	1.1%
Years of independent practice		
0–1	5	5.7%
2–3	33	37.5%
4–5	21	23.9%
5+	29	33.0%

### Confidence and Comfort in Independent Practice

The majority of surgeons (80/88—92.0%) felt that the operating skills they gained during their residency training were sufficient and appropriate for independent practice. However, 60/88 surgeons (68.2%) were not comfortable in their abilities to handle the business aspect of practice and 48/88 surgeons (54.5%) were not confident in coding and billing their services. Although 75/88 surgeons (85.2%) felt comfortable in functioning in an attending’s role, 53/88 surgeons (60.2%) believed that a transition to independent practice program would be beneficial to recent surgical graduates (refer to Table 2 for more details).

**Table 2.** Confidence and Comfort Levels of Participants (n=88) With Regard to Independent Practice.

Question	Definitely not confident (%)	Somewhat not confident (%)	Neutral (%)	Somewhat confident (%)	Extremely confident (%)	Missing (%)
Do you feel that the operating skill gained during your training is sufficient and appropriate for independent practice?	0	2.3	5.8	60.9	31.0	1.1
When you started independent practice, how confident did you feel in your surgical skills and competencies?	2.3	5.8	24.1	52.9	14.9	1.1
How confident are you in successfully managing and running an outpatient clinic?	1.2	2.3	14.9	37.9	43.7	1.1
How confident and comfortable are you in handling the business aspects of practice?	16.1	25.3	26.4	27.6	4.6	1.1
How confident are you in coding and billing your services?	4.6	20.7	28.7	36.8	9.2	1.1
How confident and comfortable are you in keeping up with latest research and practicing evidence-based medicine?	1.2	8.1	34.5	51.7	4.6	1.1
How comfortable and confident are you in functioning in an attending’s role?	0	2.3	11.5	49.4	36.8	1.1
How comfortable and confident are you in your operative decision-making as an independent practitioner?	0	0	5.8	57.0	37.2	2.2
How comfortable and confident are you in your clinical decision-making as an independent practitioner?	0	0	4.6	60.9	34.5	1.1
Do you feel you are adequately prepared to transition from a resident/fellow to an attending role?	0	4.6	19.5	51.7	24.1	1.1
How comfortable do you feel about resolving conflicts within your healthcare team in independent practice?	4.7	9.3	18.6	55.8	11.6	2.2
Do you feel a transition to independent practice program would be beneficial to recent surgical graduates?	3.5	12.6	23.0	41.4	19.5	1.1

### Perceptions of Burnout

Many surgeons (40.2%) from our study identified as having experienced burnout currently or in the past. The majority (85/88—96.6%) perceived no commitment in their workplaces to prevent burnout; 87/88 surgeons (98.9%) claimed they did not receive any formal training to prevent burnout and 82/88 surgeons (93.2%) felt that change was needed to address burnout in their workplace (refer to Table 3 for more details).

**Table 3.** Perceptions of Burnout Among Surgeons (n = 88).

Question	None (%)	Little (%)	Neutral (%)	Some (%)	More than needed (%)	Missing (%)
How much support do you feel you have at work?	3.5	18.4	27.6	43.7	6.9	1.1
How much personal support (family, friends, etc.) do you feel you have?	1.2	8.1	20.7	47.1	23.0	1.1
How much commitment do you perceive there is in your workplace to prevent burnout?	58.1	23.3	15.1	3.5	0	2.2
How taboo is the discussion of burnout and risk factors for burnout at your workplace?	8.1	17.4	24.4	33.7	16.3	2.2
How much training to prevent burnout do you perceive there to be in your workplace?	62.8	27.9	8.1	1.2	0	2.2
How much more needs to be done to address burnout in your workplace?	0	4.7	25.6	22.1	47.7	2.2
How frequently do you have to take time off work for leisure/vacation?						
Every month	n=5 (5.8%)					
Every 3 months	n=44 (50.6%)					
Every 6 months	n=31 (35.6%)					
Every 12 months	n=4 (4.6%)					
Every 12+ months	n=3 (3.5%)					
Missing	n=1 (1.1%)					
Do you perceive yourself as experiencing burnout?						
Yes, currently	n=16 (18.4%)					
Yes, previously	n=22 (25.3%)					
Maybe	n=37 (42.5%)					
Definitely not	n=15 (17.2%)					
Missing	n=1 (1.1%)					

### Maslach Burnout Inventory Scores

Over half of respondents (51/88—58.0%) met the criteria for burnout; 26 surgeons (29.6%) had high DP and 47 surgeons (53.4%) had high EE. Only 11 (12.5%) surgeons had low PA. The sensitivity analysis demonstrated no impact on burnout classification based on assigning missing question components related to EE or DP any score value between 0 and 6 (refer to Tables 4, 5).

**Table 4.** Maslach Burnout Inventory Scores (n = 88 Respondents).

MBI component	n	Percentage of total respondents (%)
Emotional exhaustion		
Low	18	20.5
Medium	23	26.1
High	47	53.4
Depersonalization		
Low	30	34.1
Medium	32	36.4
High	26	29.6
Personal accomplishment		
Low	11	12.5
Medium	29	33.0
High	48	54.6

**Table 5.** Bivariate Analysis of Demographic and Survey Characteristics to the Presence of Burnout.

	Not burnout (n=37)	Burnout (n=51)	*P*-value
Gender			.60
Male	17 (38.6%)	27 (61.4%)	
Female	19 (44.2%)	24 (55.8%)	
Age			.87
25–35	10 (43.5%)	13 (56.5%)	
36–65+	27 (41.5%)	38 (58.5%)	
Marital status			.48
Single/separated/divorced	8 (50.0%)	8 (50.0%)	
Relationship/common law/married	29 (40.3%)	43 (59.7%)	
Children			.49
None	15 (46.9%)	17 (53.1%)	
1 or more	22 (39.3%)	34 (60.7%)	
Years of independent practice			.67
0–3	15 (39.5%)	23 (60.5%)	
4–5+	22 (44.0%)	28 (56.0%)	
Surgical specialty			.69
General	14 (38.9%)	22 (61.1%)	
Subspecialty	22 (43.1%)	29 (56.9%)	
Do you feel that the operating skill gained during your training is sufficient and appropriate for independent practice?			1.00
Definitely not confident–neutral	3 (37.5%)	5 (62.5%)	
Somewhat confident–extremely confident	34 (42.5%)	46 (57.5%)	
How confident are you in successfully managing and running an outpatient clinic?			.53
Definitely not confident–neutral	6 (35.3%)	11 (64.7%)	
Somewhat confident–extremely confident	31 (43.7%)	40 (56.3%)	
How confident and comfortable are you in handling the business aspects of practice?			.72
Definitely not confident–neutral	26 (43.3%)	34 (56.7%)	
Somewhat confident–extremely confident	11 (39.3%)	17 (60.7%)	
How confident are you in coding and billing your services?			.43
Definitely not confident–neutral	22 (45.8%)	26 (54.2%)	
Somewhat confident–extremely confident	15 (37.5%)	25 (62.5%)	
How confident and comfortable are you in keeping up with latest research and practicing evidence-based medicine?			.86
Definitely not confident–neutral	16 (41.0%)	23 (59.0%)	
Somewhat confident–extremely confident	21 (42.9%)	28 (57.1%)	
How comfortable and confident are you in functioning in an attending’s role?			.78
Definitely not confident–neutral	5 (38.5%)	8 (61.5%)	
Somewhat confident–extremely confident	32 (42.7%)	43 (57.3%)	
How comfortable and confident are you in your operative decision-making as an independent practitioner?			**.02**
Definitely not confident–neutral	0 (0%)	7 (100%)	
Somewhat confident–extremely confident	37 (45.7%)	44 (54.3%)	
How comfortable and confident are you in your clinical decision making as an independent practitioner?			.07
Definitely not confident–neutral	0 (0%)	5 (100%)	
Somewhat confident–extremely confident	37 (44.6%)	46 (55.4%)	
Do you feel you are adequately prepared to transition from a resident/fellow to an attending role?			**.03**
Definitely not confident–neutral	5 (22.7%)	17 (77.3%)	
Somewhat confident–extremely confident	32 (48.5%)	34 (51.5%)	
How comfortable do you feel about resolving conflicts within your healthcare team in independent practice?			.46
Definitely not confident–neutral	11 (36.7%)	19 (63.3%)	
Somewhat confident–extremely confident	26 (44.8%)	32 (55.2%)	
Do you feel a transition to independent practice program would be beneficial to recent surgical graduates?			.15
Definitely not confident–neutral	18 (51.4%)	17 (48.6%)	
Somewhat confident–extremely confident	19 (35.9%)	34 (64.2%)	
How much support do you feel you have at work?			**.005**
None–neutral	12 (27.3%)	32 (72.7%)	
Some–more than needed	25 (56.8%)	19 (43.2%)	
How much personal support (family, friends, etc.) do you feel you have?			.12
None–neutral	8 (29.6%)	19 (70.4%)	
Some–more than needed	29 (47.5%)	32 (52.5%)	
How much commitment do you perceive there is in your workplace to prevent burnout?			1.00
None–neutral	36 (42.4%)	49 (57.7%)	
Some–more than needed	1 (33.3%)	2 (66.7%)	
How taboo is the discussion of burnout and risk factors for burnout at your workplace?			.13
Not at all–little	7 (29.2%)	17 (70.8%)	
Neutral–completely taboo	30 (46.9%)	34 (53.1%)	
How much training to prevent burnout do you perceive there to be in your workplace?			.42
None–neutral	36 (41.4%)	51 (58.6%)	
Some–more than needed	1 (100%)	0 (05)	
How much more needs to be done to address burnout in your workplace?			.23
None–little	4 (66.7%)	2 (33.3%)	
Neutral–lots of change	33 (40.2%)	49 (59.8%)	
How frequently do you have to take time off work for leisure/vacation?			.13
Every month–every 3 months (1)	25 (51.0%)	24 (49.0%)	
Every 6 months–every 12+ months	12 (34.3%)	23 (65.7%)	
Do you perceive yourself as experiencing burnout?			**.03**
Currently or previously	10 (28.6%)	25 (71.4%)	
Maybe or definitely not	27 (51.9%)	25 (48.1%)	

### Association of Demographics and Survey Characteristics With Burnout

There was no difference in burnout by gender, age, marital status, having children, years of independent practice, or by general surgical sub-specialty (Table 5). Factors associated with burnout included lack of confidence in operative decision-making as an independent practitioner (*P*=.02), lack of confidence in preparation to transition from a resident/fellow to an attending role (*P*=.03), lack of support at work (*P*=.005), and self-perception of currently or previously experiencing burnout (*P*=.03). Of the 35/87 surgeons (40.2%) from our study who perceived themselves as being burnt out, 25 surgeons (71.5%) met the criteria for burnout based on the MBI. Contrarily, of the 52/87 surgeons who were either unsure or not confident about experiencing burnout, 25 surgeons (48%) met burnout criteria.

The multivariable logistic regression model evaluating the association between demographic and survey characteristics and burnout is shown in Table 6. The overall model was significant (omnibus likelihood ratio χ^2^=12.3, *P*=.002) with 69.8% discriminant ability. A lack of support at work was associated with increased odds of having burnout (OR 4.33, 95% CI 1.68–11.20, *P*=.003), as was feeling that the discussion of burnout in the workplace is taboo (OR 3.05, 95% CI 1.02–9.13, *P*=.05).

**Table 6.** Multivariable Logistic Regression Analysis of the Association Between Survey Characteristics and Burnout.

Predictor	Adjusted odds ratio (95% CI)	Test statistic	*P*-value
Omnibus likelihood ratio (χ2 (df), *P*-value)	–	12.3 (2)	.002^a^
Support at work (none–neutral vs some–more than needed)	4.33 (1.68–11.20)	9.17	.003^a^
Discussion of burnout in the workplace is taboo (neutral–completely taboo vs not at all–little)	3.05 (1.02–9.13)	3.96	.05^a^

^a^ Statistically significant association.

## Discussion

Over half of the surgeons from our study were classified with burnout based on their responses to the MBI-HSS, although many did not identify or recognize this. This is in line with findings by Bucholz et al^24^ from a national survey of 4136 American general surgery residents. Although, our data did not highlight any sex-based differences in burnout rates, likely due to a small sample size, a higher prevalence amongst female surgeons has been clearly demonstrated by various authors.^13,25-27^ Furthermore, in the national survey of American general surgery residents conducted by Elmore et al, those who voluntarily left their training programs cited the lack of a mechanism to discuss their personal and professional concerns without fear of reprisal as a contributing factor for burnout and their decision to change careers. Our data corroborate the aforementioned finding and warrant further implementation of institutional interventions to raise awareness as well as prevent and address burnout in the workplace setting.^12,28^

Our study also found that early career general surgeons did not receive sufficient training in easing their transition to independent practice. Of note, De Montbrun et al^29^ showed that experiences during this transitional period are crucial in the growth and professional development of recently trained attending staff. Strikingly, while the majority of surgeons in our study felt confident with their technical skills and ability to operate in an attending’s role, only a minority was confident in their ability to successfully manage and run an outpatient clinic independently. Similar to our findings, Klingensmith et al^30^ have demonstrated a strong desire and need from general surgery residents in the United States for training on surgical practice administration such as coding and reimbursement, patient billing, and taxes. Hashimoto et al^31^ have also pointed toward lack of increased resident autonomy as being one of the factors hindering a smooth transition to independent practice.

This study represents the largest study on burnout among general surgeons at the start of their surgical careers. Although much focus has been placed on facilitating the transitions in other training periods (eg, from medical school to residency), no study to our knowledge has focused on the later end of the surgical training and early career period. Similar to a surgical boot camp for medical students entering surgery training, perhaps residency and fellowship programs may address their final year trainees to ensure they are adequately equipped to transition into independent practice. The American College of Surgeons has recognized this and focused efforts toward creating transition to practice programs to better equip residents for a smoother transition.^11,32^ However, long-term outcomes and utility of such curricula need to be further assessed.^33,34^ Wakeam et al has also suggested a practice-sharing model for surgeons early in their career.^35^ In this model, senior surgeons close to their retirement may mentor and train the incoming new graduate for a few years, which may help alleviate some of their stress.

A limitation of our study is the relatively small sample size of 88 surgeons across Canada. This may call into question the study’s external validity and generalizability of our findings. Although the sample size is smaller than desired, we did have a diverse population representative of many training programs. Furthermore, our study included both general surgeons as well as subspecialty surgeons practicing in both community and academic settings. Although our survey data illustrate the various types of subspecialty vs general surgeons, we do not know the distribution of the participants based on the type of practice setting (community or academic). This variable may have provided further context to our findings as these practice settings may certainly yield different issues and potential stressors for participants. A final limitation of the study, as with any survey study, is that the value of the collected data solely depends on how accurately and truthfully the participants responded to questions in the survey. In general, survey studies assume the data collected to be legitimate and reflective of the participants’ true beliefs; however, this might not always be the case. Larger studies focusing on the aforementioned drawbacks are warranted.

Our findings provide some insight into the factors contributing to burnout amongst the recently graduated general surgeon population. Since burnout adversely effects physician–patient interactions and is associated with higher economic costs (as a result of higher absenteeism, job turnover, and quality control issues), the goal in preventing or mitigating burnout is to improve not only physicians’ quality of life but also patient care.^36,37^ Future directions for research include focusing on at risk populations to identify measures necessary to reduce burnout and ease the transition of surgical graduates to independent practices.

## Conclusion

Our data showed high prevalence of burnout amongst recently graduated general surgeons across Canada. During the transition phase to independent practice, although most surgeons felt satisfied with their surgical skills, they were not confident in their managerial and administrative skills required to run a successful independent practice. Addressing these issues in surgical training programs may be beneficial for future surgeons.
